# Effects of Adolescent Intermittent Alcohol Exposure on the Expression of Endocannabinoid Signaling-Related Proteins in the Spleen of Young Adult Rats

**DOI:** 10.1371/journal.pone.0163752

**Published:** 2016-09-23

**Authors:** Francisco Javier Pavón, Eva María Marco, Mariam Vázquez, Laura Sánchez, Patricia Rivera, Ana Gavito, Virginia Mela, Francisco Alén, Juan Decara, Juan Suárez, Elena Giné, José Antonio López-Moreno, Julie Chowen, Fernando Rodríguez-de-Fonseca, Antonia Serrano, María Paz Viveros

**Affiliations:** 1 Unidad Gestión Clínica de Salud Mental, Instituto de Investigación Biomédica de Málaga (IBIMA), Hospital Regional Universitario de Málaga-Universidad de Málaga, Málaga, Spain; 2 Departamento de Fisiología (Fisiología Animal II), Facultad de Biología, Universidad Complutense, Madrid, Spain; 3 Departamento de Biología Celular, Facultad de Psicología, Universidad Complutense, Madrid, Spain; 4 Servicio de Pediatría y Endocrinología Pediátrica, Hospital Infantil Universitario Niño Jesús, Instituto de Investigación Sanitaria del Hospital Universitario de La Princesa (IP), Madrid, Spain; 5 Centro de Investigación Biomédica en Red de la Fisiopatología de la Obesidad y Nutrición (CIBERobn) Instituto de Salud Carlos III, Madrid, Spain; National Institutes of Health, UNITED STATES

## Abstract

Intermittent alcohol exposure is a common pattern of alcohol consumption among adolescents and alcohol is known to modulate the expression of the endocannabinoid system (ECS), which is involved in metabolism and inflammation. However, it is unknown whether this pattern may have short-term consequences on the ECS in the spleen. To address this question, we examined the plasma concentrations of metabolic and inflammatory signals and the splenic ECS in early adult rats exposed to alcohol during adolescence. A 4-day drinking in the dark (DID) procedure for 4 weeks was used as a model of intermittent forced-alcohol administration (20%, v/v) in female and male Wistar rats, which were sacrificed 2 weeks after the last DID session. First, there was no liver damage or alterations in plasma metabolic parameters. However, certain plasma inflammatory signals were altered according to sex and alcohol exposition. Whereas fractalkine [chemokine (C-X3-C motif) ligand 1] was only affected by sex with lower concentration in male rats, there was an interaction between sex and alcohol exposure in the TNF-α and interleukin-6 concentrations and only female rats displayed changes. Regarding the mRNA and protein expression of the ECS, the receptors and endocannabinoid-synthesizing enzymes were found to be altered with area-specific expression patterns in the spleen. Overall, whereas the expression of the cannabinoid receptor CB_1_ and the nuclear peroxisome proliferator-activated receptor PPARα were lower in alcohol-exposed rats compared to control rats, the CB_2_ expression was higher. Additionally, the N-acyl-phosphatidylethanolamine-specific phospholipase D expression was high in female alcohol-exposed rats and low in male alcohol-exposed rats. In conclusion, intermittent alcohol consumption during adolescence may be sufficient to induce short-term changes in the expression of splenic endocannabinoid signaling-related proteins and plasma pro-inflammatory cytokines in young adult rats with a strong sexual dimorphism. The potential impact of these alterations in early adulthood remains to be elucidated.

## Introduction

Alcohol is the most commonly used addictive drug in our society, and its consumption is increasing among young people and adolescents, becoming a major public health concern. Intermittent alcohol exposure is a common pattern of alcohol consumption among adolescents, which can lead eventually to heavy episodic drinking (called binge drinking) [1]. Adolescent alcohol exposure has immediate health effects related to alcoholic intoxication (e.g., fatigue, headache and sleeping problems) but also long-term negative physical and mental health consequences from young adulthood because adolescence is a period of heightened sensitivity (e.g., coronary and circulatory diseases, anxiety and mood and personality disorders) [1,2]. Moreover, there is a robust association between age at first drink and the risk of substance use disorders in adulthood, primarily alcoholism [3].

Alcohol consumption is linked to the immune system affecting the expression of several inflammatory signals (e.g., cytokines and chemokines) and other signaling systems closely related to inflammatory responses such as the endocannabinoid system (ECS). Whereas numerous studies have demonstrated that heavy episodic or binge drinking is able to modulate the production of inflammatory mediators [4,5], acute and moderate alcohol consumption have also an inhibitory effect on the immune response by down-regulating the production and release of pro-inflammatory cytokines [6,7,8,9]. In addition, endocannabinoids and other fatty acid derivatives (e.g., N-palmitoylethanolamine) are potent anti-inflammatory agents that can exert their effects through regulation of cytokine production and release [10,11]. Several studies have demonstrated the relevance of the ECS in mediating the behavioral and pharmacological effects of alcohol [12].

The ECS includes the cannabinoid receptors, the endogenous ligands (endocannabinoids) and the enzymatic machinery for their synthesis and inactivation. The main endocannabinoids are N-arachidonoyl-ethanolamine (anandamide, AEA) and 2-arachidonoyl-glycerol (2-AG), which can be directly produced on demand from membrane precursors. In this case, endocannabinoids are primarily synthesized by N-acyl-phosphatidylethanolamine-specific phospholipase D (NAPE-PLD) and diacyl-glycerol lipase (DGL) for AEA and 2-AG, respectively. Regarding the main degradative enzymes, AEA is inactivated by fatty acid amide hydrolase (FAAH) and 2-AG by monoacyl-glycerol lipase (MAGL). However, there are a growing number of additional pathways and enzymes involved into the endocannabinoid metabolism that are being investigated [13]. The cannabinoid receptors are mainly G protein-coupled receptors but also other targets have been proposed for endocannabinoids and their congeners (e.g., the transient receptor potential vanilloid type-1 and peroxisome proliferator-activated receptor). Two canonical cannabinoid receptors have been characterized and cloned, CB_1_ and CB_2_, which are found throughout the body. However, although CB_1_ receptors are highly expressed in brain, CB_2_ receptors have been classically located in immune cells [14,15].

During inflammatory processes, the activation of CB_2_ receptors inhibits the production of chemokines (e.g., chemokine (C-C motif) ligand [CCL2]) and pro-inflammatory cytokines (e.g., interleukin-1 beta [IL-1β], IL-6 and tumor necrosis factor alpha [TNF-α]), and increases the secretion of anti-inflammatory cytokines, such as interleukin-10 (IL-10) [10]. These immunosuppressive and anti-inflammatory effects were initially observed using the plant-derived cannabinoid delta-9-tetrahidrocannabinol (THC) [16] and subsequently with endocannabinoids [17,18]. Although numerous studies have demonstrated an interaction between alcohol consumption and the ECS at the level of the central nervous system, there is very little information concerning how alcohol consumption affects the ECS at the immune system level. In this regard, a recent study has shown a functional role of the ECS on monocyte-derived dendritic cells exposed to alcohol with higher levels of CB_2_ genes (Cnr2) and IL-1β production [19]. However, there are no studies focused on the impact of alcohol exposure in the ECS in the spleen, considering this organ has a critical role in the immune response.

The present study in rats was designed to investigate the effects on the splenic ECS-related proteins in young adult rats after an intermittent alcohol exposure during adolescence because early adulthood appears to be a critical period to consolidate addictive behaviors. Because remarkable sex differences have been described regarding the consequences of acute ethanol intoxication on the immune response in rats [20], female and male animals were studied. Additionally, we have analyzed relevant inflammatory mediators and metabolic parameters in the plasma and liver of these animals to detect inflammatory processes.

## Materials and Methods

### Animals and Ethical Statement

Experiments were conducted in the offspring of Wistar rats purchased from Harlan Laboratories (Rossdorf, Germany) in our animal facilities at Universidad Complutense (registration # EX08-UCS). A total of 32 male and female animals from 4 litters were employed in this experiment. On the day of birth, postnatal day (PND) 0, the litters were adjusted and sex-balanced to 8 pups per dam (4 males and 4 females). The animals were left undisturbed until weaning at PND 22; thereafter, animals were housed in pairs of sibling of the same sex in standard Plexiglas cages (50×25×17.5 cm) in a room with temperature (22±1°C) and humidity (55±5%) control on a reversed 12-h dark/light cycle (lights on at 20:00 h). The rats had *ad libitum* access to food (diet A04/A03; SAFE, Augy, France) and fresh tap water, except during exposure to alcohol. All animals were monitored daily for health status. The prototypic adolescence in rats was chosen using a conservative age range (PND 28–42) during which animals of both sexes would be expected to exhibit adolescent-typical neurobehavioral characteristics and because during this interval, vaginal opening occurs in females and marked increases are seen in the number of maturing spermatids in the seminiferous tubules in males [21]. All procedures were approved by the Laboratory Animal Use Committee of the Universidad Complutense (CEA-UCM 78/2012) in strict adherence with the European Directive 2010/63/EU on the protection of animals used for scientific purposes and Spanish regulations (Real Decreto 53/2013 and Ley 32/2007). All efforts were made to minimize unnecessary suffering and distress.

### Drinking in the Dark Model

**Fig 1:**
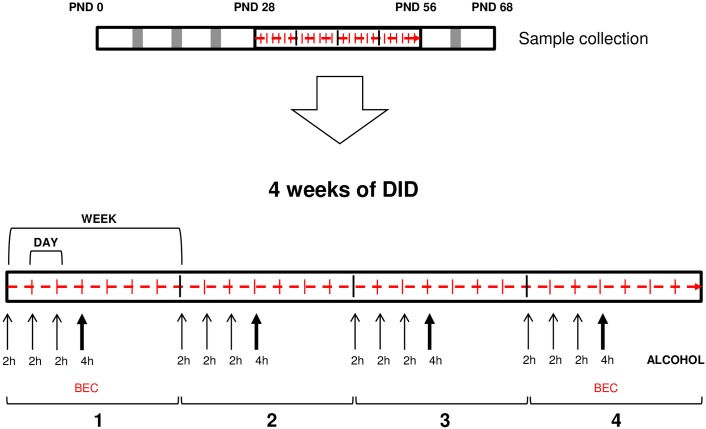
Schematic representation of the 4-day drinking in the dark (DID) procedure for 4 weeks.

As shown in Fig 1, a 4-day drinking in the dark (DID) procedure was used as a model of intermittent forced-alcohol administration [22]. Adolescent rats (PND 28, body weigh = 84.5±15.6 g) were randomly assigned to the alcohol or control group (8 female and 8 male rats per group) in individual cages and alternating the placement of these cages from each experimental group. During 4 weeks, animals were exposed to a single bottle of alcohol (20%, v/v) or tap water for 4 consecutive days 1 h after beginning the dark cycle. On days 1–3 access to the bottles was limited to a 2-h session, whereas on day 4 the exposure time was extended to a 4-h session. This 4-day DID procedure was followed by 3 days of abstinence (only water was available) each week [23]. Bottles were weighed before and after each drinking session.

### Determination of Blood Alcohol Concentration

Rats were tail-bled 90 min after the 4-h session on the first and last week of alcohol or water (DID) exposure for blood ethanol concentration (BEC). Blood samples were collected in Microvette CB300 tubes containing EDTA (Fisher Scientific, Madrid, Spain) and centrifuged at 2,000xg for 15 min. Plasma was extracted and assayed for ethanol content using the EnzyChrom ethanol assay kit (Bioassay Systems, Hayward, CA, USA). All determinations were performed in duplicate.

### Sample Collection

Two weeks after the last DID exposure, rats were euthanized at 12:00 h (i.e., 4 h after lights on) in a room provided with low-intensity white noise. Rats were sacrificed by decapitation (PND 68) and blood, liver and spleen samples were collected. Blood was centrifuged (2,000xg for 15 min) and the plasma kept for further analysis. Spleen samples were divided into 2 pieces: one piece was snap-frozen in liquid N_2_ and stored at -80°C until mRNA and protein analyses; and the other piece was fixed in 4% formaldehyde in 0.1 M phosphate-buffered saline (PBS) for 24 h and embedded in paraffin for histology and immunohistochemistry. Additionally, Liver samples were also frozen and stored at -80°C.

### Plasma Biochemical Analysis

The following metabolites were measured in the plasma samples: glucose, triglycerides, cholesterol, high-density lipoprotein cholesterol (HDL-C), uric acid, urea, creatinine, glutamate-oxoloacetate transaminase (GOT), glutamate-pyruvate transaminase (GPT) and ɣ-glutamyl transpeptidase (GGt). These metabolites were analyzed using commercial kits according to the manufacturer’s instruction in a Hitachi 737 Automatic Analyzer (Hitachi, Tokyo, Japan).

### Multiplex Immunoassay Analysis

A Bio-Plex Suspension Array System 200 (Bio-Rad Laboratories, Hercules, CA, USA) was used to quantify cytokine and chemokine concentrations in plasma with a MILLIPLEX^®^ MAP (Multi-analyte panels) Rat Cytokine/Chemokine Magnetic Bead Panel (EMD Millipore, Darmstadt, Germany). This method of analysis is based on the Luminex technology and a rat cytokine/chemokine 6-plex panel was used to simultaneously detect the following analytes: TNF-α; IL-1β; IL-6; IL-10; CCL2 and the chemokine (C-X_3_-C motif) ligand 1 (CX_3_CL1). The measurements of these analytes in plasma were performed following the manufacturer’s instructions [24]. Data are expressed as pg of protein/mL of plasma.

### Liver Fat and Triglyceride Content

Total lipids were extracted from frozen liver samples with chloroform-methanol (2:1, v/v) according to the Bligh and Dyer method [25]. After 2 centrifugation steps (2,800xg for 10 min, 4°C), the lower phase containing lipids was extracted. N_2_ was used to dry each sample, and the liver fat content was expressed as percentage of tissue weight. The lipid extracts with known weight were separated by one-dimensional high-performance thin layer chromatography using hexane-diethylether-acetic acid (80:20:1, v/v/v) as the solvent system. After separation, the lipid spots corresponding to triglycerides were scraped from silica gel plates (Merck, Darmstadt, Germany). Total triglycerides were extracted from silica and quantified in relation to the total lipids after drying each sample with N_2_ [26].

### RNA Isolation and qRT-PCR Analysis

Total RNA was extracted from spleen samples using Trizol Reagent (Gibco BRL Life Technologies, Baltimore, MD, USA). To ensure the purity of the mRNA sequences and exclude proteins and molecules smaller than 200 nucleotides, RNA samples were isolated with an RNeasy Minelute Cleanup Kit (Qiagen, Hilden, Germany). The total mRNA concentrations were quantified using a spectrophotometer (Nanodrop 1000 Spectrophotometer, Thermo Scientific, Rochester, NY, USA) to ensure A260/280 ratios of 1.8 to 2.0. Reverse transcription was performed using the Transcriptor Reverse Transcriptase kit and random hexamer primers (Transcriptor RT, Roche Diagnostic, Mannheim, Germany). Quantitative real-time reverse-transcription polymerase chain reaction (qRT-PCR) was performed using an ABI PRISM^®^ 7300 Real-Time PCR System (Applied Biosystems, Foster City, CA, USA) and the FAM dye label format for the TaqMan^®^ Gene Expression Assays (Applied Biosystems, Foster City, CA, USA). Melting curves analysis was performed to ensure that only a single product was amplified. We analyzed various housekeeping genes and selected β-actin gene (Actb) as the most suitable according to its homogeneity. The relative quantification was calculated using the ΔΔCt method and normalized to the female control group. Primers for the qRT-PCR (S1 Table) were obtained based on TaqMan^®^ Gene Expression Assay search tool for rats (https://bioinfo.appliedbiosystems.com/genome-database/gene-expression.html).

### Protein Extraction and Western Blot Analysis

The spleen samples were disrupted in lysis buffer supplemented with a cocktail of protease inhibitors (cOmplete tablets, Roche Diagnostic, Mannheim, Germany). The suspension was shaken for 2 h at 4°C and centrifuged at 20,800xg for 15 min at 4°C, recovering the soluble fraction below the fat ring. Protein concentration was determined by Bradford protein assay. Protein extracts were diluted 1∶1 in 2X sample buffer containing DTT and boiled for 5 min before submitting to SDS-PAGE. Samples (50μg of total proteins each) were resolved in gradient SDS-PAGE gels (Bio-Rad Laboratories, Madrid, Spain) and blotted onto nitrocellulose membranes (Bio-Rad Laboratories, Madrid, Spain). Membranes were blocked in TBS-T (50 mM Tris-HCl, pH 7.6; 200 mM NaCl, and 0.1% Tween-20) with 2% BSA for 1 h. Specific proteins were detected by incubation in TBS-T, 2% BSA for 2 h with the corresponding primary antibodies purchased from Abcam (Cambridge, UK): polyclonal rabbit anti-CB_1_ (1:200 dilution; Cat No. ab23703), anti-CB_2_ (1:200 dilution; Cat No. ab3561), anti-PPARα (1:500 dilution; Cat No. ab8934), anti-NAPE-PLD (1:200 dilution; Cat No. ab95397) and anti-β actin (1:1,000 dilution; Cat No. ab8227). After extensive washing in TBS-T, anti-rabbit-HRP conjugated secondary antibody (Promega, Madison, MI, USA) was added for 1 h. Membranes were subjected to extensive washings in TBS-T and the specific protein bands were revealed using the enhanced chemiluminiscence detection system (Santa Cruz, Dallas, TX, USA), in accordance with the manufacturer’s instructions, and images were visualized in an Autochemi-UVP Bioimaging System. Bands were quantified by densitometric analysis performed by ImageJ software (Rasband, W.S., ImageJ, U.S., National Institutes of Health, Bethesda, MA, USA, http://imagej.nih.gov/ij, 1997–2012). Values were expressed in relation to β-actin.

### Histological Exploration and Immunohistochemical Analysis

Spleen sections embedded in paraffin were cut by microtome (5-μm thick), mounted on D-polylysinated glass slides, deparaffinized in xylene, and stained with haematoxylin and eosin for histological evaluation. Paraffined spleen blocks were cut into 5 μm-thick sections using a Microm HM 325 microtome (MICROM, Walldorf, Germany) and organized on glass slides. Sections were dewaxed, and washed several times with Tris-buffered saline (TBS pH 7.8), and incubated in 3% hydrogen peroxide in TBS for 30 min in the dark at room temperature in order to inactivate endogenous peroxidase. After 3 washes in TBS for 5 min, antigen retrieval was achieved by incubating in sodium citrate (pH 6.0) for 15 min at 80°C. A background blocker solution containing 10% donkey serum, 0.3% triton X-100 and 0.1% sodium azide was used to incubate the sections for 2 h, which was followed by 24-h incubation at room temperature with the following primary antibodies: rabbit anti-CB_1_ (1:50 dilution; Cat No. ab23703; Abcam, Cambridge, UK), anti-CB_2_ (1:50 dilution; Cat No. ab3561; Abcam, Cambridge, UK), anti-PPARα (1:50 dilution; Cat No. 20R-PR021; Fitzgerald, North Acton, MA, USA) and anti-NAPE-PLD (1:50 dilution; Cat No. 10306; Cayman, Ann Arbor, MI, USA). Sections were washed 3 times with TBS, incubated in a biotinylated donkey anti-rabbit IgG (Amersham, Barcelona, Spain) diluted 1:100 for 2 h, washed again in TBS, and incubated in ExtrAvidin peroxidase (Sigma, St. Louis, MO, USA) diluted 1:500 in darkness at room temperature for 1 h. After 3 washes in TBS in darkness, we revealed immunolabeling with 0.05% diaminobenzidine, 0.05% nickel ammonium sulfate and 0.03% H_2_O_2_ in PBS. All steps were performed by gentle agitation at room temperature. Sections were counterstained with haematoxylin. Then, they were dehydrated in ethanol, cleared in xylene, and coverslipped with Eukitt mounting medium (O. Kindler, Freiburg, Germany). Digital high-resolution microphotographs of spleen sections were taken at 4x, 10x and 40x magnification under the same conditions of light and brightness/contrast by an Olympus BX41 microscope equipped with an Olympus DP70 digital camera (Olympus Iberia, Barcelona, Spain).

### Statistical Analysis

All data for graphs and tables are expressed as the mean ± SEM. The different experiments included 8 animals per group (alcohol female, alcohol male, control female and control male group) according to the assay. The statistical analysis of mRNA levels and protein concentrations was conducted using two-way analysis of variance (ANOVA) [factors: adolescent alcohol exposure (alcohol/control) and sex (female/male)] followed by *post hoc* tests with *Bonferroni* corrections for multiple comparisons when there were significant interactions. A *p*-value less than 0.05 was considered statistically significant. All statistical analyses were performed using the Graph-Pad Prism version 5.04 software (GraphPad Software, San Diego, CA, USA).

## Results

### Blood Alcohol Concentrations

Growing concentrations of ethanol were found in the blood of alcohol rats during the intermittent exposure following a 4-day DID procedure for 4 weeks. Two hours after the last DID session of the first week (PND 31), the average BEC was as follows: 11.97±4.28 mg/dL in female rats and 10.99±1.18 mg/dL in male rats. After the last DID session of the fourth week (PND 52), the BEC was 29.34±3.92 mg/dL in female rats and 28.26±2.20 mg/dL in male rats. As expected, a two-way ANOVA revealed a main effect of time of alcohol exposure (number of DID sessions) on the BEC (F_1,28_ = 30.06, *p*<0.001) of rats with no effect of sex or interaction between both factors. In accordance with the BEC data, the average ethanol intake for PND 31 and PND 52 was as follows: 0.74±0.18 g/kg and 1.12±0.19 g/kg in female rats; and 0.69±0.18 g/kg and 1.03±0.09 g/kg in male rats.

### Effects of Adolescent Intermittent Alcohol Exposure on Plasma Parameters and Hepatic Fat Content

To characterize the metabolic state of young adult rats exposed to repeated cycles of alcohol during adolescence, we evaluated the plasma concentrations of relevant parameters related to glucose and lipid metabolism, as well as to toxicity. In addition, we analyzed the lipid content in liver to evaluate whether this pattern of alcohol consumption produced hepatic damage taking sex into consideration. As shown in Table 1, no main effects or interaction of adolescent alcohol exposure and sex were observed on the expression of these plasma parameters and the hepatic fat accumulation.

**Table 1:** Biochemical parameters in the plasma and liver of rats exposed to alcohol during adolescence.

	Female		Male	
	Control	Alcohol	Control	Alcohol
**Glucose (mg/dL)**	165.13±8.64	159.88±7.88	174.88±6.00	167.28±3.82
**Triglycerides (mg/dL)**	208.63±26.50	246.00±31.65	246.00±31.65	255.50±28.45
**Cholesterol (mg/dL)**	88.63±6.78	85.13±4.64	97.50±6.93	95.75±4.73
**HDL-C (mg/dL)**	38.93±2.18	38.48±2.46	45.33±3.73	41.99±2.91
**Urea (mg/dL)**	54.61±2.41	52.16±3.46	54.74±2.53	58.10±7.12
**Uric acid (mg/dL)**	2.54±0.19	2.56±0.16	2.38±0.20	2.22±0.16
**Creatinine (mg/dL)**	0.48±0.03	0.48±0.05	0.49±0.05	0.53±0.05
**GOT (U/L)**	339.88±34.46	285.64±19.88	262.18±22.93	287.38±12.63
**GPT (U/L)**	83.95±4.67	84.29±6.49	93.34±7.83	88.07±2.64
**GGt (U/L)**	6.10±0.69	4.81±0.51	6.05±1.09	6.48±0.45
**Fat content in liver (% tissue)**	4.07±0.12	3.94±0.14	4.05±0.13	3.77±0.14
**Triglycerides in liver (% tissue)**	0.94±0.08	0.91±0.07	0.89±0.07	0.87±0.06

Abbreviations: HDL-C = High-density lipoprotein cholesterol; GPT = Glutamate-pyruvate transaminase; GOT = Glutamate-oxaloacetate transaminase; GGt = Gamma glutamyl transpeptidase. The data are expressed as the mean ± SEM (n = 8 animals per group).

### Effects of Adolescent Intermittent Alcohol Exposure on Plasma Chemokine and Cytokine Concentrations

**Fig 2:**
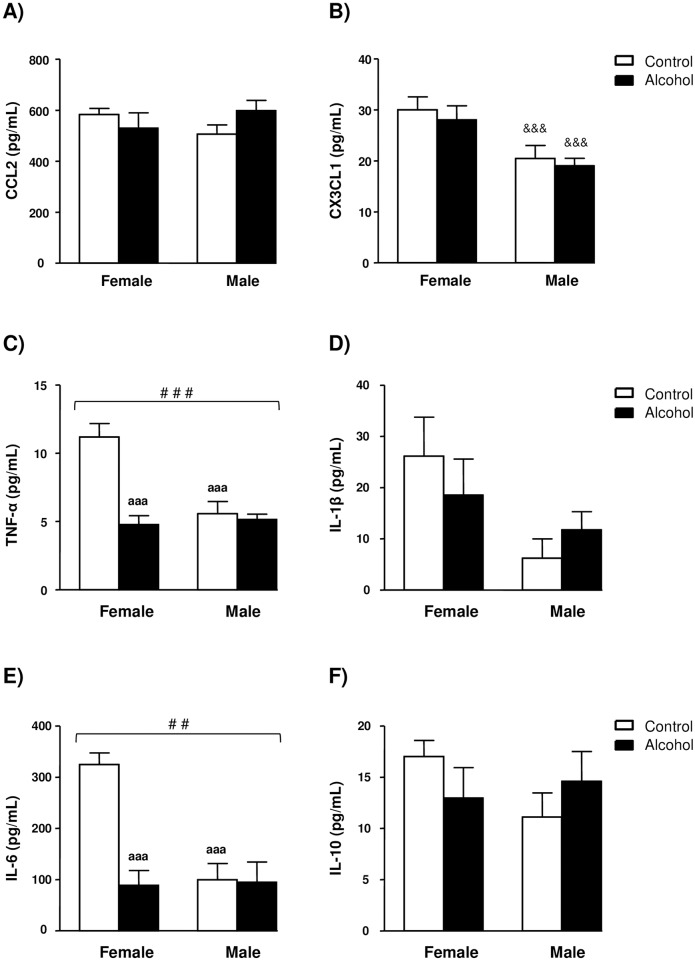
Plasma concentrations of chemokines in rats exposed to alcohol during adolescence and control rats grouped by sex. (A) CCL2 (pg/mL); (B) CX_3_CL1 (pg/mL); (C) TNF-α (pg/mL); (D) IL-1β (pg/mL); (E) IL-6 (pg/mL); and (F) IL-10 (pg/mL). The bars represent means ± SEM. The bars represent means ± SEM. The data were analyzed by two-way ANOVA and significant main effects or interactions between factors were indicated as follows: (^&&&^) denotes significant main effect of *sex*; (^##^) p<0.01 and (^###^) p<0.001 denote significant interaction between *adolescent alcohol exposure* and *sex*; (^aaa^) p<0.001 denotes significant differences compared to female control rats after *post hoc* tests.

Plasma concentrations of the chemokines CCL2 and CX_3_CL1and the cytokines TNF-α, IL-1β, IL-6 and IL-10 are depicted in Fig 2.

A two-way ANOVA of plasma CCL2 concentrations revealed no main effects or interaction between alcohol exposure and sex (Fig 2A). In contrast, there was a main effect of sex on the plasma CX_3_CL1 concentrations (F_1,28_ = 14.19, *p*<0.001), but no effect of alcohol exposure or interaction. As Fig 2B indicates, CX_3_CL1 concentrations were significantly lower in male rats than female rats.

In relation to TNF-α, although there were significant main effects of alcohol exposure (F_1,28_ = 19.99, *p*<0.001) and sex (F_1,28_ = 11.83, *p* = 0.002) on TNF-α concentrations, the analysis revealed a significant interaction between both factors (F_1,28_ = 15.22, *p*<0.001). The interaction overrides both main effects because high TNF-α concentrations were only observed in female control rats as Fig 2C shows. In fact, *post hoc* pairwise comparisons showed that TNF-α concentrations were significantly lower in male control rats (*p*<0.001) and female alcohol-exposed rats (*p*<0.001) compared to female control rats.

IL-1β concentrations were not affected by alcohol exposure or sex and there was no interaction. However, there was a trend toward higher IL-1β concentrations in female rats than male rats as Fig 2D illustrates.

Similar to TNF-α, plasma IL-6 concentrations were significantly affected by alcohol exposure (F_1,20_ = 14.32, *p* = 0.002) and sex (F_1,20_ = 11.90, *p* = 0.004) but also a significant interaction (F_1,20_ = 13.15, *p* = 0.003) that overrides both main factors. As shown in Fig 2E, IL-6 concentrations were found to be higher than the rest of experimental groups. Thus, plasma IL-6 concentrations were lower in male control rats (*p*<0.001) and female alcohol-exposed rats (*p*<0.001) than female control rats. Some samples were OOR for IL-6 (8 samples and their duplicates).

Finally, there were no main effects or interaction between alcohol exposure and sex on plasma IL-10 concentrations (Fig 2F).

### Histology of the Spleen After Adolescent Intermittent Alcohol Exposure

**Fig 3:**
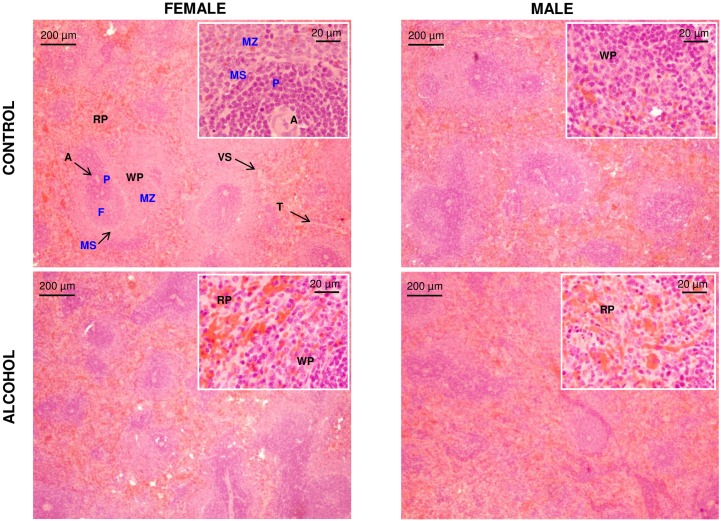
Representative images after histological analysis of spleen sections using haematoxylin and eosin stain. Spleen sections are shown at 4x and 40x magnification. Abbreviations: A = Central artery; F = Follicle; MS = Marginal sinus region; MZ = Marginal zone; P = Periarteriolar lymphoid sheath; RP = Red pulp; T = Trabeculus; VS = Venous sinus; WP = White pulp.

Before analyzing the mRNA and protein expression of receptors and enzymes related to endocannabinoid metabolism, histological studies with haematoxylin and eosin stain allowed to examine morphological aspects of the spleen (Fig 3).

Representative spleen sections at 4x magnification showed no remarkable differences in the morphology of the two main splenic compartments, the white pulp (WP) and the red pulp (RP), and type of cells when the experimental groups were compared. Details on type of cells are shown in images at 40x magnification. Thus, the WP consists of lymphatic tissue and the densely packed heterochromatin of the lymphocyte nuclei was responsible for the purple color (haematoxylin staining). We observed several aggregations of lymphocytes (B and T-cells) surrounding the central arteries. The abundance of erythrocytes in the RP was easily detectable for the red color (eosin staining). In addition to erythrocytes, other cells (e.g., reticular and dendritic cells) and a relatively smaller amount of lymphatic cells were also observed in the splenic cords of the RP.

### Effects of Adolescent Intermittent Alcohol Exposure on mRNA Expression of Endocannabinoid Signaling-Related Proteins in the Spleen

**Fig 4:**
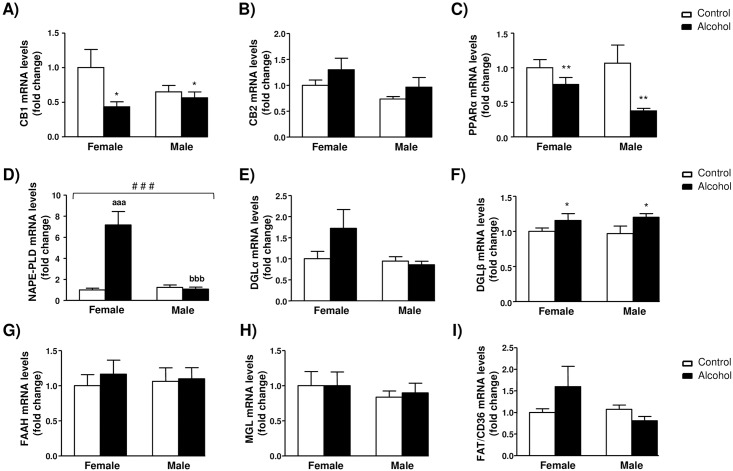
Relative mRNA expression of endocannabinoid signaling-related proteins in the spleen of rats exposed to alcohol during adolescence and control rats grouped by sex. (A) CB_1_ mRNA levels (fold change); (B) CB_2_ mRNA levels (fold change); (C) PPARα mRNA levels (fold change); (D) NAPE-PLD mRNA levels (fold change); (E) DGLα mRNA levels (fold change); (F) DGLβ mRNA levels (fold change); (G) FAAH mRNA levels (fold change); (H) MGL mRNA levels (fold change); and (I) FAT/CD36 mRNA levels (fold change). The bars represent means ± SEM. The data were analyzed by two-way ANOVA and significant main effects or interaction between factors was indicated as follows: (*) p<0.05 and (**) p<0.01 denote significant main effect of *adolescent alcohol exposure*; (^###^) p<0.001 denotes significant interaction between *adolescent alcohol exposure* and *sex*. (^aaa^) p<0.001 denotes significant differences compared to female control rats after *post hoc* tests; (^bbb^) p<0.001 denotes significant differences compared to female alcohol-exposed rats after *post hoc* tests.

The mRNA expression of receptors and enzymes related to the ECS was analyzed by qRT-PCR in the spleen. Fig 4 shows the relative mRNA expression of cannabinoid CB_1_ and CB_2_ receptors, the peroxisome proliferator-activated receptor alpha (PPARα), endocannabinoid-synthesizing enzymes (N-acyl phosphatidylethanolamine-phospholipase D [NAPE-PLD] and diacylglicerol lipases [DGLα and DGLβ]), and endocannabinoid-degrading enzymes (fatty acid amide hydrolase [FAAH] and monoacylglycerol lipase (MGL)]. Additionally, we measured the mRNA expression of the fatty acid translocase FAT/CD36 because this enzyme regulates fatty acid mobilization.

A two-way ANOVA revealed a significant main effect of alcohol exposure on the CB_1_ receptor mRNA levels (F_1,28_ = 5.656, *p* = 0.024) and alcohol-exposed rats had lower levels of CB_1_ receptor mRNA than control rats (Fig 4A). Although the CB_2_ receptor mRNA levels appear to be increased in alcohol-exposed rats, and male rats had higher CB_2_ levels than female rats, the effects of alcohol exposure or sex did not reach statistical significance (Fig 4B). Similar to CB_1_ receptor, PPARα mRNA levels were only affected by alcohol exposure (F_1,28_ = 9.354, *p* = 0.005) and lower PPARα mRNA levels were observed in alcohol-exposed rats relative to control rats (Fig 4C).

Statistical analysis of NAPE-PLD mRNA levels revealed main effects of alcohol exposure (F_1,28_ = 20.13, *p*<0.001) and sex (F_1,28_ = 19.10, *p*<0.001) and most importantly a significant interaction between these factors (F_1,28_ = 22.34, *p*<0.001). The disordinal interaction was because of higher mRNA levels in female alcohol-exposed rats than the rest of experimental groups (Fig 4D). *Post hoc* pairwise comparisons indicated that the levels of NAPE-PLD mRNA were significantly higher in female alcohol-exposed rats compared to female control rats (*p*<0.001) and male alcohol-exposed rats (*p*<0.001). Regarding DGLα and DGLβ mRNA levels, we found statistical differences. Whereas there was no significant effect of alcohol exposure or sex on the expression of DGLα mRNA (Fig 4E), we found a main effect of alcohol exposure (F_1,28_ = 4.471, *p* = 0.041) on the DGLβ mRNA levels and higher levels were observed in alcohol-exposed rats (Fig 4F).

The mRNA expression of the hydrolytic enzymes (FAAH and MGL) and FAT/CD36 were also measured. However, there were no main effects or interaction between alcohol exposure and sex on the levels of FAAH (Fig 4G), MAGL (Fig 4H) and FAT/CD36 (Fig 4I) mRNA.

### Effects of Adolescent Intermittent Alcohol Exposure on Protein Expression of Endocannabinoid Signaling-Related Proteins in the Spleen

**Fig 5:**
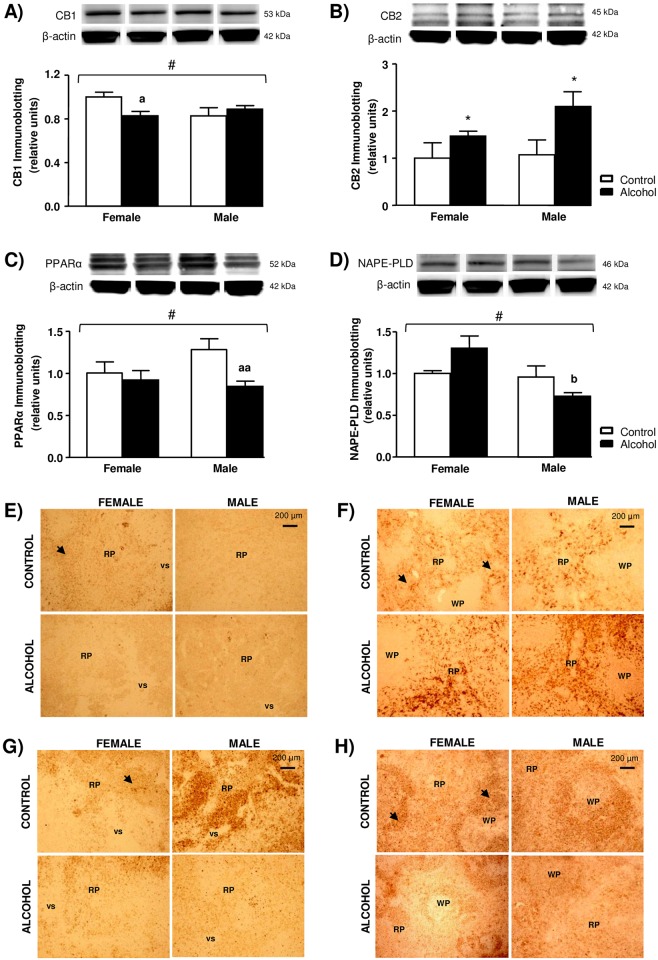
Expression of CB_1_, CB_2_, PPARα and NAPE-PLD proteins in the spleen of rats exposed to alcohol during adolescence and control rats grouped by sex. Relative protein expression and representative immunoblots of: (A) CB_1_ protein (fold change); (B) CB_2_ protein (fold change); (C) PPARα protein (fold change); and (D) NAPE-PLD protein (fold change). Representative immunohistochemical images at 4x magnification of: (E) CB_1_ protein; (F) CB_2_ protein; (G) PPARα protein; and (H) NAPE-PLD protein. The bars represent means ± SEM. The data were analyzed by two-way ANOVA and significant main effects or interaction between factors was indicated as follows: (*) p<0.05 denotes significant main effect of *adolescent alcohol exposure*; (^#^) p<0.05 denotes significant interaction between *adolescent alcohol exposure* and *sex*. (^a^) p<0.05 and (^aa^) p<0.01 denote significant differences compared to the respective control rats after *post hoc* tests; (^b^) p<0.05 denotes significant differences compared to female alcohol-exposed rats after *post hoc* tests. The black arrows indicate positive cells. Abbreviations: RP = Red pulp; VS = Venous sinus; WP = White pulp.

Based on mRNA data, the protein expression of receptors and the endocannabinoid-synthesizing enzyme NAPE-PLD were analyzed by Western blot in the spleen. Complementarily, the same proteins were studied using immunohistochemistry. Fig 5 shows the relative protein expression by immumoblotting and representative immunohistochemical images at 40x magnification.

A two-way ANOVA revealed no significant main effects of alcohol exposure and sex on CB_1_ receptor expression but there was a significant interaction (F_1,20_ = 5.379, *p* = 0.031). *Post hoc* comparisons indicated that CB_1_ receptor expression was significantly lower in female alcohol-exposed rats than female control rats (*p*<0.05) (Fig 5A). With respect to CB_2_ receptor protein, there was a significant main effect of alcohol exposure (F_1,20_ = 5.041, *p* = 0.036) and alcohol-exposed rats had higher CB_2_ receptor expression than control rats in both sexes (Fig 5B). PPARα receptor was significantly affected by alcohol exposure (F_1,20_ = 7.526, *p* = 0.012) and there was a significant alcohol exposure × sex interaction (F_1,20_ = 6.470, *p* = 0.019). As shown in Fig 5C, the main effect of alcohol was only the case for male rats and *post hoc* comparisons showed that male alcohol-exposed rats had lower protein expression than male control rats (*p*<0.01) and no differences between female rats were found.

Regarding the presence of NAPE-PLD protein by western blot, there was a main effect of sex (F_1,20_ = 7.922, *p* = 0.011) but also a significant interaction between alcohol exposure and sex (F_1,20_ = 5.520, *p* = 0.029). Thus, high protein levels in female rats only occurred for female rats exposed to adolescent alcohol displaying high protein levels (Fig 5D). In fact, *post hoc* comparisons showed significant differences in NAPE-PLD levels when female alcohol-exposed rats were compared to male alcohol-exposed rats (p<0.05).

**Fig 6:**
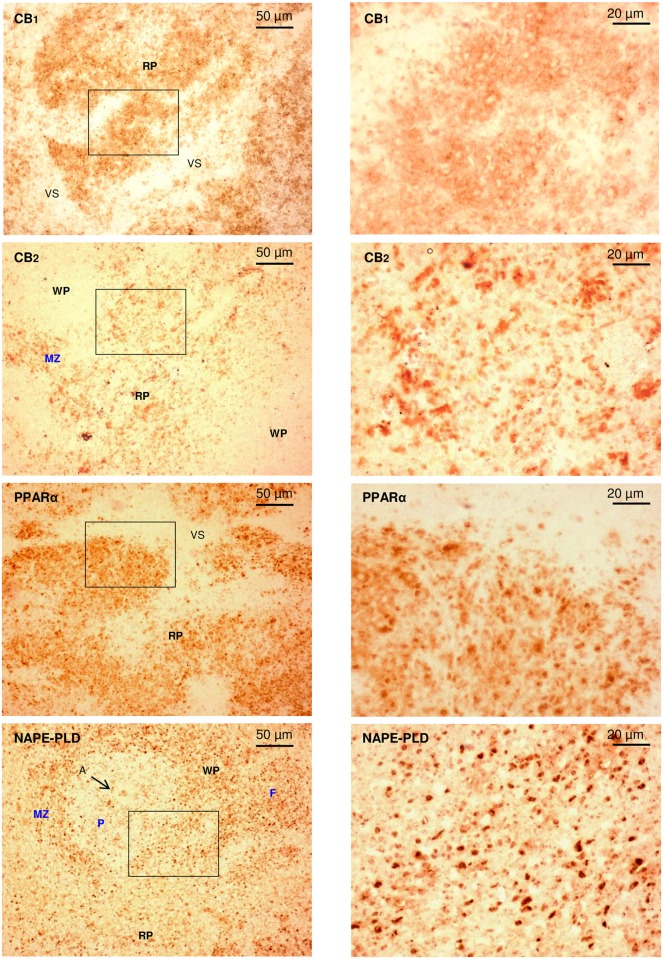
Representative immunohistochemical images of spleen sections at 10x and 40x magnification of CB_1_, CB_2_, PPARα and NAPE-PLD from female control rats. The squares indicate the areas of 40x magnification. Abbreviations: A = Central artery; F = Follicle; MZ = Marginal zone; P = Periarteriolar lymphoid sheath; RP = Red pulp; VS = Venous sinus; WP = White pulp.

Overall, the immunohistochemical studies of CB_1_, CB_2_, PPARα and NAPE-PLD in spleen sections agreed with the western blot results and representative images at 4x magnification were included (Fig 5E–5H). However, the density of these proteins in the spleen was no homogeneous and we found an area-specific distribution. Thus, all receptors were primarily expressed in the RP while NAPE-PLD was expressed in the WP. Exploratory visualization of different sections concluded that there were no changes in the localization of the staining for these proteins after comparing the experimental groups and, consequently, we selected the female control group to show the detailed distribution of these proteins using immunohistochemical images at higher magnifications (Fig 6).

Whereas CB_1_ and PPARα appeared in splenic cords surrounding venous sinuses, CB_2_ was found in splenic cords surrounding the WP but also in the marginal zone (MZ) of the WP. In contrast to receptors, NAPE-PLD was strongly expressed in the WP, especially in follicles that may contain germinal centers and outer periarteriolar lymphoid sheaths (PALS).

## Discussion

Relevant findings from the present study in young adult rats exposed to alcohol during early adolescence can be summarized as follows. First, the DID procedure of adolescent intermittent alcohol exposure was not associated with the induction of fatty liver or changes in biochemical parameters related to lipid and glucose metabolism in the plasma of rats. Second, the plasma concentrations of TNF-α and IL-6 were found to be different in female alcohol-exposed rats compared to female control rats with no differences in male rats. Third, the mRNA and protein expression of endocannabinoid signaling-related proteins in the spleen were found to be significantly different in alcohol-exposed rats relative to control rats, primarily in receptors (with opposite changes in CB_1_ and CB_2_ expression) and synthetic enzymes with sex differences. Furthermore, we found an area-dependent density variation of these altered endocannabinoid signaling-related proteins in the spleen.

However, we are aware of the differences in the duration and evolution of adolescence in male and female rats and the exploratory nature of the present study.

### Moderate Alcohol Consumption

We used a 4-day DID procedure for 4 weeks in rats to mimic an intermittent ethanol exposure during adolescence [23] and this procedure produced a rise over time in alcohol consumption, which was reflected in an increase in the average BEC. However, these concentrations did not indicate binge drinking (defined as heavy episodic drinking within a BEC of 80 mg/dL or higher) but a moderate drinking [27]. It is important to indicate that the blood samples were collected 2 h after finishing the DID-session with a length of 4 h, and this delay may influence the BEC values.

Because early adulthood appears to be a critical period to consolidate addictive behaviors and begin symptoms of alcohol use disorders [28,29], we examined endocannabinoid signaling-related proteins in the spleen and metabolic and inflammatory signals in the plasma of female and male rats, 2 weeks after finishing the intermittent alcohol exposition. As expected, these animals did not display alcoholic fatty liver disease [30] and there was no increase in hepatic triglyceride content or changes in plasma concentrations of transaminases (GPT and GOT) and GGt. Once hepatic damage was discarded, we tested the effects of adolescent alcohol exposure on circulating inflammatory signals and main components of the endocannabinoid system in the spleen.

### Inflammatory Signals in the Plasma

In the present study, we measured plasma concentrations of relevant chemokines and anti- and pro-inflammatory cytokines, which have been reported to be altered after alcohol exposure [31,32]. Growing evidence has demonstrated naturally occurring sex differences in immune responses and inflammatory mediators and it is thought that fluctuations in estrogen may alter immune cell function by affecting cytokine and chemokine production [33]. Overall, we found sex-differences in the plasma concentrations of these inflammatory signals in control rats but there were clear interactions with adolescent alcohol exposition in certain pro-inflammatory cytokines.

TNF-α and IL-6 levels were significantly lower in alcohol-exposed rats but this was only the case for female rats, which suggest that alcohol use during adolescence may exert a short-term inhibitory effect on the expression of these cytokines in female rats displaying a higher susceptibility to alcohol effects. Regarding the rest of signals, there was a strong sexual dimorphism in the expression of CX_3_CL1 so that female rats displayed higher levels than male rats. Similarly, previous studies in rats reported greater CX_3_CL1 expression in mesenteric arterial bed [34] and periaqueductal gray [35] in female than male rats.

### Endocannabinoid Signaling-Related Proteins in the Spleen

Histological studies indicated no changes in the morphology of splenic sections in the experimental groups, identifying both main internal compartments (i.e., the RP and the WP) and other structures and type of cells.

The ECS and alcohol have been widely studied in the CNS but also in the liver [36,37]. In fact, numerous studies have reported that the ECS is dysregulated in alcoholic fatty liver, presumably increasing the endocannabinoid concentrations and CB_1_ receptor activity [36,37]. However, the relationship between the ECS and alcohol abuse has been less studied in other relevant tissues such as the spleen, which plays a critical role in the immune system.

#### Receptors

Because there is a lack of literature about the expression of cannabinoid receptors in the spleen in association with alcohol exposure, our data provide the first evidence for an opposite response of CB_1_ and CB_2_ in the spleen.

CB_1_ receptors were first thought to be expressed exclusively in the brain but are expressed to a lesser degree in peripheral organs. Thus, the splenic CB_1_ expression has been previously reported, with a significant amount of CB_1_ mRNA in the spleen capsule [38]. Our findings are consistent with previous studies conducted in rat cerebral areas (i.e., amygdala and hippocampus) using intermittent alcohol exposures, which reported decreased CB_1_ receptor mRNA levels during early abstinence [39,40]. Even more interesting, a recent study with human monocyte-derived dendritic cells from alcohol users showed lower levels of CB_1_ gene (Cnr1) compared to non-users but also higher levels of CB_2_ gene (Cnr2) [19]. Unlike CB_1_ receptors, CB_2_ receptors are more prominently localized in the immune system and peripheral tissues and the anti-inflammatory and antinociceptive effects of CB_2_ receptor activation have received a great deal of attention [41]. Previous data suggest that CB_2_ receptors play an important role in alcohol addiction but these studies have been performed in the brain of rodent models using pharmacological and genetic approaches [42]. Regarding peripheral tissues, although CB_2_ has been reported to protect against alcoholic liver disease [43] there are no data about the association between alcohol exposure and the splenic CB_2_ receptor.

Regarding PPARα, this nuclear receptor has been studied primarily in the liver and numerous studies show that ethanol consumption impairs fatty acid catabolism in liver by blocking PPARα-mediated responses [44]. Here, we observed no changes in hepatic and plasma triglycerides 2 weeks after alcohol exposure but there was lower PPARα expression including mRNA and protein. Recently, we have demonstrated that circulating levels of N-oleoylethanolamine (OEA) are altered in rodents exposed to alcohol and abstinent patients [45,46]. This N-acylethanolamine is a natural ligand for PPARα and a short abstinence from alcohol induces increased OEA levels in the plasma [46], which may induce a down-regulation of PPARα.

The immunohistochemical data showed a primary presence of these receptors in the RP of the spleen, although CB_2_ was also found in the MZ. The RP is a blood filter composed of splenic cords and venous sinuses accompanied of fibers and several types of cells such as erythrocytes, granulocytes, macrophages, mononuclear cells and lymphocytes [47]. Additional studies using specific markers and antibodies will be needed to identify the type(s) of cell expressing receptors such as CB_1_ and PPARα in the splenic cords surrounding the venous sinuses and the WP compartment. As commented, CB_2_ was strongly expressed in the MZ, a highly transited area that receives large amounts of blood from the general circulation with a high presence of resident metallophilic macrophages and B-cells [47,48]. This interface area is involved in the presentation of antigens from the systemic circulation to lymphocytes and CB_2_ may participate in this antigen processing, taking in to account the high expression of CB_2_ in immune cells [15]. In particular, CB_2_ has been reported to be critical for the homing and retention on B-cells from the MZ in rodent models and profound deficiencies in this area have been observed in mice deficient in CB_2_ [49].

#### NAPE-PLD

NAPE-PLD is the biosynthetic enzyme of N-acylethanolamines (e.g., AEA and OEA) and its expression has been found to be higher in female alcohol-exposed rats than the rest of subgroups, particularly compared to male alcohol-exposed rats. Previously, we reported that a continuous 2-week alcohol exposure with liquid diet (10% ethanol, w/v) of adult male rats is associated with an increase in the mRNA expression of NAPE-PLD which is not found in the male rats of present study. However, it is important to indicate that the mentioned study was conducted in the amygdala and there is no data about the effect on female rats. Previous molecular studies in human neuroblastoma cells have shown that ethanol exposure (100 mM) for 72 h is associated with increased formation of AEA and NAPE-PLD [50], and therefore the high expression of NAPE-PLD in female alcohol-exposed rats may be linked to high levels of N-acylethanolamines which will have to be elucidated.

As indicated previously, the immunohistochemical studies showed that the presence of NAPE-PLD was primarily detected in the WP, especially in the follicles and external layers of the PALS (close to the MZ). The WP is responsible for the lymphoid activity and initiates immune responses to circulatory antigens [47]. In particular, whereas outer PALS and follicles are mainly populated by B-cells with different sizes and macrophages, inner PALS is populated by (CD4+) T-cells and migrating B-cells. It has been reported that arrest and proliferation of B cells in the outer PALS are required for the subsequent formation of germinal centers within follicles [51]. The localization of B-cells in the spleen was concordant with the expression of NAPE-PLD but this finding will have to be confirmed by means of specific marker.

### Endocannabinoid Signaling and Inflammatory Signals

Endocannabinoids have been shown to exhibit immunosuppressive and anti-inflammatory effects, and the activation of cannabinoid receptors by endocannabinoids or THC inhibits the production of pro-inflammatory cytokines and chemokines and increases the secretion of anti-inflammatory cytokines [10,18]. The high expression of CB_2_ and NAPE-PLD in the spleen of female rats exposed to adolescent intermittent alcohol may be compatible with increased endocannabinoid N-acylethanolamines (e.g., AEA). Further, we have reported high plasma N-acylethanolamine levels in alcohol-addicted patients during abstinence [46], but this observation has to be cautiously considered because of the experimental differences. Following this rationale, high levels of N-acylethanolamines may be linked to low plasma concentrations of TNF-α and IL-6, which is in accordance with previous *in vitro* studies that demonstrate that an increase in the AEA tone, either directly or indirectly through its biosynthesis/degradation enzymes, induces a decrease in the release of pro-inflammatory cytokines [52,53,54]. In fact, studies conducted in cocaine users during abstinence reported that increased plasma levels of endocannabinoid N-acylethanolamines are associated with decreased levels of pro-inflammatory cytokines such as TNF-α [55,56]. Thus, alcohol-related changes in the levels of circulating cytokines and endocannabinoids may be associated to changes in the proliferation, differentiation and activation of lymphocytes (e.g., B-cells and CD4+ T cells) and other immune cells in the spleen altering the responsiveness of the immune system. Finally, it should be pointed out that our data demonstrate the existence of sexual dimorphism of the expression of splenic proteins related to the ECS and circulating inflammatory signals. Although this study was focused on the spleen, the sex differences observed in the ECS have been extensively reported [57,58,59].

Functional studies using pharmacological and genetic approaches will be needed to elucidate the role of the splenic ECS in the expression of inflammatory signals and the impact of a early alcohol exposure.

## Supporting Information

S1 Table Primers used for qRT-PCR (TaqMan^®^ Gene Expression Assays). (DOCX)
