# Supplementary material for: Effects of Adolescent Intermittent Alcohol Exposure on the Expression of Endocannabinoid Signaling-Related Proteins in the Spleen of Young Adult Rats
Source: PLoS One. 2016 Sep 23;11(9):e0163752. doi: 10.1371/journal.pone.0163752 (PMC5035052; doi:10.1371/journal.pone.0163752)
Supplement: S1 Table — (DOCX) [file pone.0163752.s001.docx]

**S1 Table. Primers used for qRT-PCR (TaqMan® Gene Expression Assays).**

| Gene symbol | Description (nomenclature) | Assay ID | GenBank accession number | Amplicon length |
| --- | --- | --- | --- | --- |
| *Actb* | actin, beta | Rn00667869_m1 | NM_031144.2 | 91 |
| *Cnr1* | cannabinoid receptor 1 | Rn02758689_s1 | NM_012784.4 | 92 |
| *Cnr2* | cannabinoid receptor 2 | Rn03993699_s1 | NM_001164142.1 | 102 |
| *Ppara* | peroxisome proliferator activated receptor alpha | Rn00566193_m1 | NM_013196.1 | 98 |
| *Lpar1* | lysophosphatidic acid receptor 1 | Rn00588435_m1 | NM_053936.3 | 67 |
| *Napepld* | N-acyl phosphatidylethanolamine phospholipase D | Rn01786262_m1 | NM_199381.1 | 71 |
| *Dagla* | diacylglycerol lipase, alpha | Rn01454304_m1 | NM_001005886.1 | 67 |
| *Daglb* | diacylglycerol lipase, beta | Rn01453770_m1 | NM_001107120.1 | 57 |
| *Faah* | fatty acid amide hydrolase | Rn00577086_m1 | NM_024132.3 | 63 |
| *Mgll* | monoglyceride lipase | Rn00593297_m1 | NM_138502.2 | 78 |
| *Cd36* | CD36 molecule (thrombospondin receptor) | Rn02115479_g1 | AF111268.1 | 122 |
